# A Rare Case of Prostatic Utricle with Crossover Vas Deferens in Children

**DOI:** 10.3390/medicina58010040

**Published:** 2021-12-27

**Authors:** Tsung-Heng Wu, Yao-Jen Hsu, Tai-Wai Chin, Yu-Wei Fu

**Affiliations:** Department of Pediatric Surgery, Changhua Christian Hospital, Changhua 500, Taiwan; wu150533@gmail.com (T.-H.W.); 181606@cch.org.tw (Y.-J.H.); 180266@cch.org.tw (T.-W.C.)

**Keywords:** prostatic utricle, laparoscopy, crossover vas deferens

## Abstract

*Background:* A prostatic utricle (PU) is an unusual pathology with most patients being asymptomatic. However, approximately 29% of patients may show lower urinary tract symptoms, recurrent urinary tract infections (UTI), postvoid dribbling, urethral discharge, epididymo-orchitis, stones, and secondary incontinence caused by urine trapping in the pouch and urinary retention. The standard treatment is through surgical resection, but it is only offered to patients with symptoms. *Case summary:* We report a case involving a six-year-old boy with congenital hypothyroidism and penoscrotal hypospadias who had previously undergone onlay urethroplasty for the proximal shaft, chordee release, orchidopexy for bilateral undescended testis, and laparoscopic herniorrhaphy for left inguinal hernia. However, the patient later evolved the repetition of UTI and right epididymo-orchitis. Cyclic voiding cystourethrography confirmed the presence of a cystic lesion communicating with the prostatic urethra from the utricle. The PU was then excised laparoscopically. The utricle was identified posterior to the bladder, and insertions of the vas deferens crossover into the utricle were detected by laparoscopy. The post-procedure course was uneventful. *Conclusions:* Laparoscopic resection of PUs offers a better exposure field, improved wound appearance, complete resection, and reduces the incidence of complications. During laparoscopy, the PU was clearly distinguished from the bladder or other pelvic organs. An incidental finding of vas deferens crossover has rarely been reported. A combined cystoscopy and laparoscopy for PU resection is executable, safe, and valid in this patient population.

## 1. Introduction

A prostatic utricle (PU), also called the Müllerian duct cyst, is a cyst that arises from remnants of the Müllerian duct. PU is an unusual pathology, and almost all patients are asymptomatic. However, approximately 29% of patients may show lower urinary tract symptoms, recurrent urinary tract infections (UTIs), postvoid dribbling, urethral discharge, epididymo-orchitis, stones, and secondary incontinence caused by urine trapping in the pouch and urinary retention. The main standard for management is through surgical resection, but it is only provided to patients with symptoms [1].

## 2. Case Presentation

A six-year-old boy with congenital hypothyroidism and penoscrotal hypospadias had previously undergone onlay urethroplasty for proximal shaft, chordee release, orchidopexy for bilateral undescended testis, and laparoscopic herniorrhaphy for left inguinal hernia.

**Figure 1:**
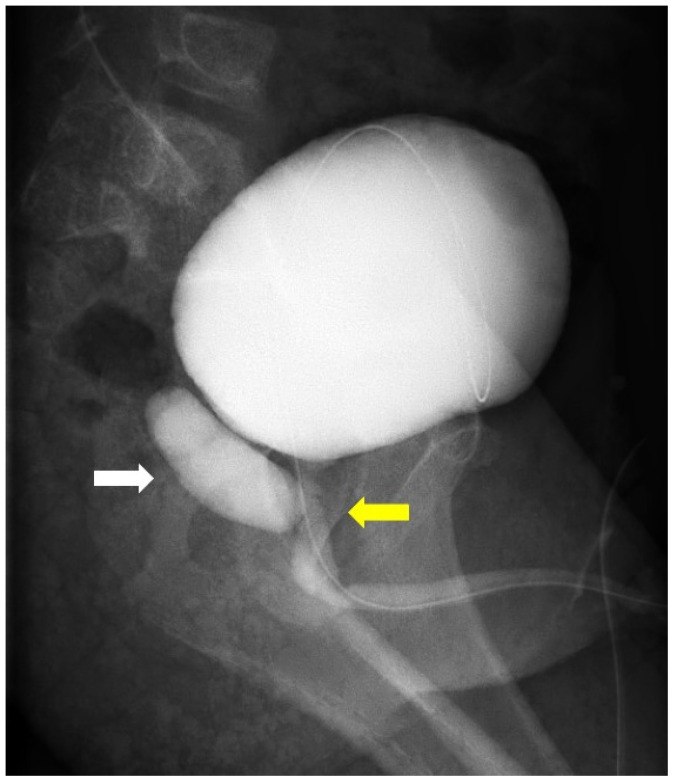
Cyclic voiding cystourethrography. A cystic lesion that communicates with the prostatic urethra (yellow arrow) posteriorly is suspected to be a large prostatic utricle (write arrow).

However, the patient later evolved repetition of UTI and right epididymo-orchitis. Finally, cyclic voiding cystourethrography confirmed the presence of a cystic lesion communicating with the prostatic urethra from the utricle (Figure 1). The PU was then excised laparoscopically.

**Figure 2:**
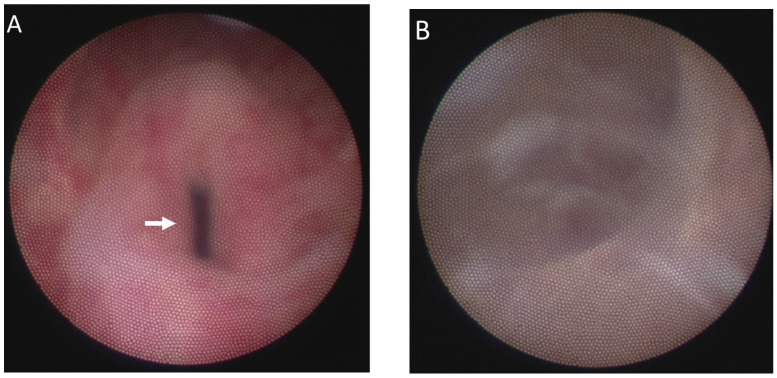

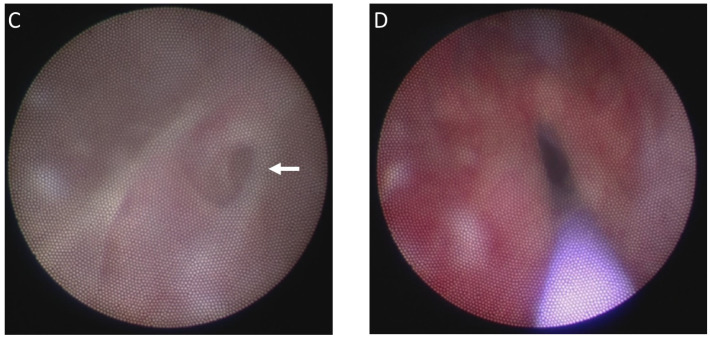
(**A**) Dilated opening of the utricle (arrow). (**B**) A cystoscope was inserted into the dilated prostatic utricle. (**C**) Left-sided dilated opening of the ejaculatory duct (arrow). (**D**) Guidewire was inserted into the utricle for further identification under laparoscopic surgery.

First, urethrocystoscopy was performed, and a guidewire was inserted into the utricle for PU identification. A left-sided ejaculatory duct dilatation was noted (Figure 2).

**Figure 3:**
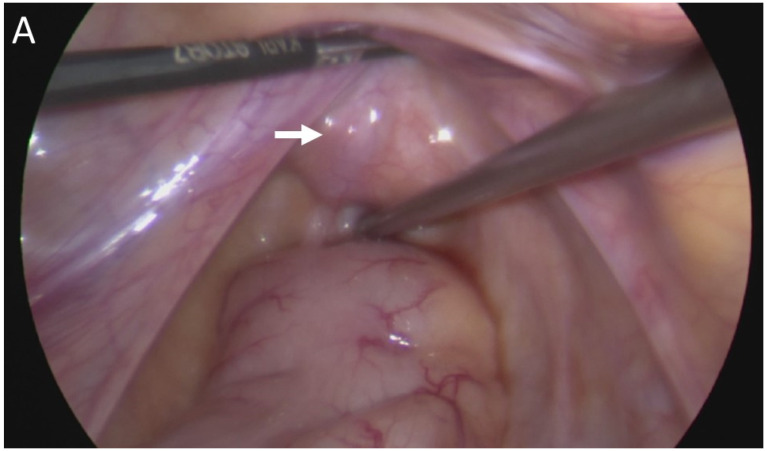

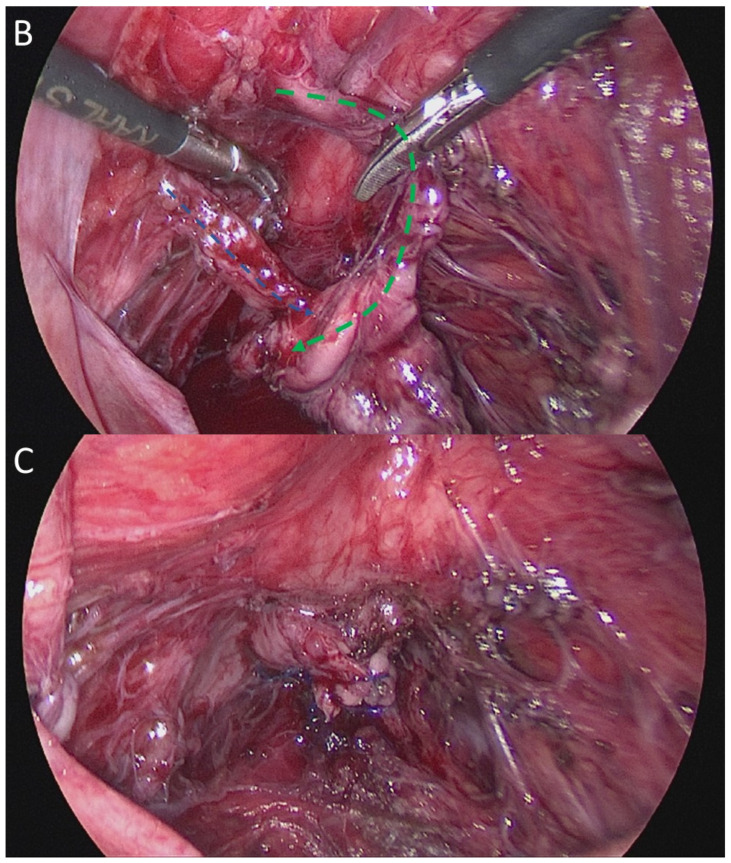
(**A**) Large utricle lesion was found between the bladder and rectum laparoscopically (arrow). (**B**) Crossover vas deferens from the right vas deferens to the left opening of the ejaculatory duct was observed (dotted green line arrow) and left vas deferens (dotted blue line arrow). (**C**) Utricle was completely resected.

Afterward, laparoscopy was performed using a 5-mm cannula via the umbilicus and two 3-mm cannula trocars on the right and left lower quadrant of the abdomen. The prostatic utricle was carefully dissected free of surrounding tissue to the pelvic floor. The utricle was identified posterior to the bladder, and insertions of the vas deferens crossover into the utricle were observed. When the prostatic utricle was completely mobilized, the utricle and vas deferens were completely resected, and then the urethra was repaired with 6-0 PDS (Figure 3).

Laparoscopic resection of the PU was successful in this patient. After the surgery, the patient recovered smoothly, with no complications and voiding difficulties, when the urethral catheter was removed on day two and then discharged. The patient visited our outpatient clinic in the first month after the operation and in the third month, then patients typically attend routine visits every 6 months at our outpatient clinic. No further UTIs or epididymo-orchitis occurred after ten months of follow-up consultation.

## 3. Discussion

A PU, also called the Müllerian duct cyst, is produced by the effect of either decreased hormonal secretion or shortage of hormone sensitivity in tissues at the stage when the urogenital plate, which the fused tips of the Müllerian duct originate from, comes into contact with the urogenital sinus, leading to PU formation [2]. Some children with disorders of sexual differentiation (DSDs), especially Persistent Mȕllerian Duct Syndrome (PMDS), have PU formation.

The PMDS is one of the DSDs categories. PMDS is a rare autosomal recessive disorder characterized by the persistence of Mȕllerian structures in a boy. In most cases, phallic development and testicular function are normal. The disorder is usually confirmed during surgery for inguinal hernia with or without cryptorchidism. Most cases are due to mutations in AMH (Anti-Müllerian hormone) or AMHR2 (Anti-Müllerian Hormone Receptor Type 2) [3]. In the case of bilateral cryptorchidism associated with a hernia and normal genital appearance, as in our case, the possibility of PMDS should be kept in mind. Although the real incidence of abnormally enlarged PUs in males is hard to evaluate, it has been reported that 14% of proximal hypospadias and 57% of perineal hypospadias occur in abnormally enlarged Pus [4]. Additionally, others have reported that as the severity of hypospadias increases, the incidence of PU increases [1,2,5]. Some reports suggest that the enlarged utricle causes an ejaculatory duct obstruction and infertility [6]. Schuhrke described a neoplastic degeneration in 3% of PUs [7], with a peak incidence at about the fourth decade of life. Therefore, early resection has been publicly accepted as a treatment option.

Several different surgical methods for PU resection have been described; however, the standard surgical method has not yet been established. Moreover, the problem of dissecting PUs deep in the pelvis and preventing infertility is another issue because these are anatomically close to the spermatic cord.

Open surgical procedures are related to the hazard of insufficient PU resection, injury to important pelvic structures, and poor exposure [1,8]. Transurethral, transvesical, transperitoneal, perineal, and posterior sagittal (transrectal or perirectal) approaches are the most common open procedures [1,8,9,10]. Although the transperitoneal approach makes it easy to explore the intrapelvic organs, it can be difficult for dissection deep in the pelvis [10]. The hazard of injury to the pudendal nerve, external sphincter, and rectum is increased under the perineal approach [10]. The transvesical approach is recommended to improve the exposure and ease of dissection; however, the trigonal function can be disturbed [10]. Therefore, it is suggested to obtain the best exposure through the posterior sagittal approach [1,10]. The disadvantage of the transrectal approach is the necessity of preoperative bowel preparation and rectal incision [1,10].

Due to the disabilities associated with open surgical procedures, McDougall et al. [11] reported the first successful laparoscopic resection of a Müllerian duct cyst with a diameter of 7 cm in 1994, and Yeung et al. put forward laparoscopic treatment in 2001 [1]. The laparoscopic resection of PUs offers a better exposure field, wound appearance, total resection, and reduces the incidence of complications [1,8,9]. Additionally, Jia et al. reported that compared with open surgery, laparoscopic surgery significantly reduced length of stay and blood loss, and shortened operation time [1].

A residual utricle stump was observed in three cases following the open excision of the PU, while no instances were observed following the laparoscopic approach [1]. Additionally, in laparoscopy, there is no need for the bladder or rectum incised.

Recently, robot-assisted laparoscopy has increased all the benefits of common laparoscopy by raising the anatomic visual image and surgical precision when dissecting within the deep pelvis [12]. However, robot-assisted laparoscopy is quite expensive and unavailable in most hospitals. Furthermore, robot-assisted laparoscopy was not suitable for our six-year-old patient.

## 4. Conclusions

A patient with a history of penoscrotal hypospadias underwent an onlay procedure, chordee release, and inguinal hernia repair presented with repeated UTIs. Differential diagnoses should include a utricle cyst. Furthermore, laparoscopic resection of the PU can be assumed as a viable option for open surgery because it supplies better visualization, simple dissection deep in the pelvis, and better wound healing.

A guidewire was put into the PU under cystoscopy. A Foley catheter was also inserted into the bladder to deflate it and differentiate the junction where the cyst neck enters the prostatic urethra, which helped avoid complications of injuring the urethra. In addition, the surgeon could clearly distinguish between the PU and the bladder or other pelvic organs under laparoscopy. An incidental finding of crossover vas deferens is rarely reported. Thus, the combination of cystoscopy and laparoscopy for PU resection is executable, safe, and valid in this patient population.
